# Research trends and hotspot analysis of sudden cardiac death in hypertrophic cardiomyopathy: A bibliometric analysis

**DOI:** 10.1097/MD.0000000000051272

**Published:** 2026-10-23

**Authors:** Zhen Chen, Fen Ai, Jinlei Li, Yu Li

**Affiliations:** a Department of Emergency Medicine, The Central Hospital of Wuhan, Tongji Medical College, Huazhong University of Science and Technology, Wuhan, China

**Keywords:** bibliometric analysis, hypertrophic cardiomyopathy, sudden cardiac death

## Abstract

**Background:**

Hypertrophic cardiomyopathy is the most common genetic heart disease and a leading cause of sudden cardiac death. This study aims to map the research landscape of Hypertrophic cardiomyopathy -related sudden cardiac death over the past 2 decades using bibliometric methods.

**Methods:**

We performed a bibliometric analysis of 712 publications retrieved from the Web of Science Core Collection (2005–2025). Using Cite Space, we examined publication trends, collaboration networks, co-citation patterns, and keyword dynamics.

**Results:**

Annual publication output rose markedly, with pronounced accelerations after the 2014 European Society of Cardiology and 2020 American heart association/American College of Cardiology guidelines. The United States led in productivity (203 publications) and network centrality (0.40), while China ranked second in output (123 publications) but exhibited limited international collaboration (centrality = 0.01). Co‑citation analysis indicated that clinical guidelines and risk‑prediction models form the field’ score knowledge base, and five of the ten most cited references were international guidelines. Keyword analysis defined 4 principal thematic clusters disease entity, clinical outcomes, imaging biomarkers, and risk stratification and documented a shift from early mechanistic studies to multimodal risk prediction, followed more recently by artificial intelligence–driven models and targeted pharmacological therapies (e.g., mavacamten).

**Conclusion:**

This study provides a systematic, data-driven overview of the knowledge structure and evolutionary trends in Hypertrophic cardiomyopathy-related sudden Cardiac death research. The findings underscore the field’ s guideline-driven character, document the rise of AI-based risk stratification, and describe a paradigm shift toward disease-modifying therapies, offering actionable insights for prioritizing future research and informing clinical decision-making.

## 1. Introduction

Hypertrophic cardiomyopathy (HCM) is the most common inherited cardiac disorder and a leading cause of sudden cardiac death (SCD).^[1]^ Since the early 21st century, risk assessment and management strategies for HCM-related SCD have evolved significantly. This progression has moved from the relatively simple evaluation of clinical risk factors outlined in the 2003 American College of Cardiology (ACC)/European Society of Cardiology(ESC) expert consensus to the formal incorporation of late gadolinium enhancement on cardiac magnetic resonance imaging as an independent risk marker in the 2014 ESC guidelines.^[2,3]^ More recently, novel pharmacological therapies (e.g., mavacamten) and artificial intelligence-driven predictive models have been explored.^[4]^ These developments marked the field’s transition toward a more precise and dynamic era of risk management.

Numerous studies have examined the mechanisms, risk assessment, and intervention strategies for HCM-related sudden cardiac death (HCM-SCD).^[1]^ The publication of the 2014 ESC and 2020 American heart association (AHA)/ACC guidelines in particular has accelerated the standardization of clinical practice and research in this field.^[3–5]^ However, a systematic analysis of the research landscape, knowledge structure, and evolutionary trends over the past 2 decades using bibliometric methods is lacking.

Bibliometric analysis involves a systematic and quantitative examination of the scientific literature. Measuring the distribution, relationships, and clustering of publications within a field is widely used to understand knowledge structures, identify emerging topics, and track developmental trends.^[6]^ Although bibliometric methods have examined diverse cardiovascular topics including heart failure,^[7]^ atrial fibrillation,^[8]^ and cardiomyopathies ^[9]^ no study has systematically mapped the knowledge domain of HCM-related SCD. Existing reviews are mainly narrative or guideline-focused and lack quantitative, structural, and evolutionary analyses. This study fills that gap by presenting the first bibliometric dissection of HCM-related sudden Cardiac death (HCM-SCD) research spanning 2 decades, with particular emphasis on collaboration patterns, intellectual turning points, and emerging frontiers.

Therefore, This study aims to characterize the publication landscape and collaborative networks in HCM-SCD research; delineate the field’ s intellectual structure and knowledge base through co-citation analysis; trace evolutionary trajectories and identify emerging hotspots using keyword co‑occurrence, burst detection, and timeline mapping; and evaluate the degree to which international clinical guidelines have shaped the field.

## 2. Materials and methods

### 2.1. Data collection

The data for this study were sourced from the Web of Science (WoS) Core Collection. The search was conducted on October 6, 2025, to ensure data consistency. A systematic search strategy was employed, utilizing a combination of topic terms, title words, AND, OR author keywords connected by Boolean operators (AND, OR) to comprehensively capture the research literature at the intersection of HCM and SCD. The specific search query was as follows: TS = (“HCM” OR “HCM”) AND TS = (OR “SCD” OR “ventricular arrhythmia” OR “cardiac arrest”) (Full search history and all variant terms are provided in Supplementary Table S1, Supplemental Digital Content 1). The inclusion criteria were as follows: publication date between January 1, 2005, and October 6, 2025, and peer-reviewed original research articles. Exclusion criteria were publications not in English and non-research items such as conference abstracts, letters, review article, meeting abstract, meta-analyses, guidelines, consensus statements, editorial material, early access, proceeding paper, and corrections. Application of these criteria yielded a final corpus of 712 publications. All retrieved records were exported in plain text format and saved as “download txt.” Each publication was screened based on inclusion and exclusion criteria. This structured approach facilitated an organized synthesis of the literature, enabling a comprehensive analysis of the current research trends and gaps. Although WoS is the predominant source for bibliometric analysis due to its high-quality citation data, this approach may overlook non-English studies and those indexed solely in regional or multidisciplinary databases, such as Scopus or PubMed. Two authors, Yu Li and Zhen Chen, independently read the title, abstract, and full text of all articles and then deleted articles that are not relevant to the topic. If there was disagreement among them, discussion or recourse was made to the third author, Fen Ai. The detailed study selection process is shown in Figure 1.

**Figure 1.**
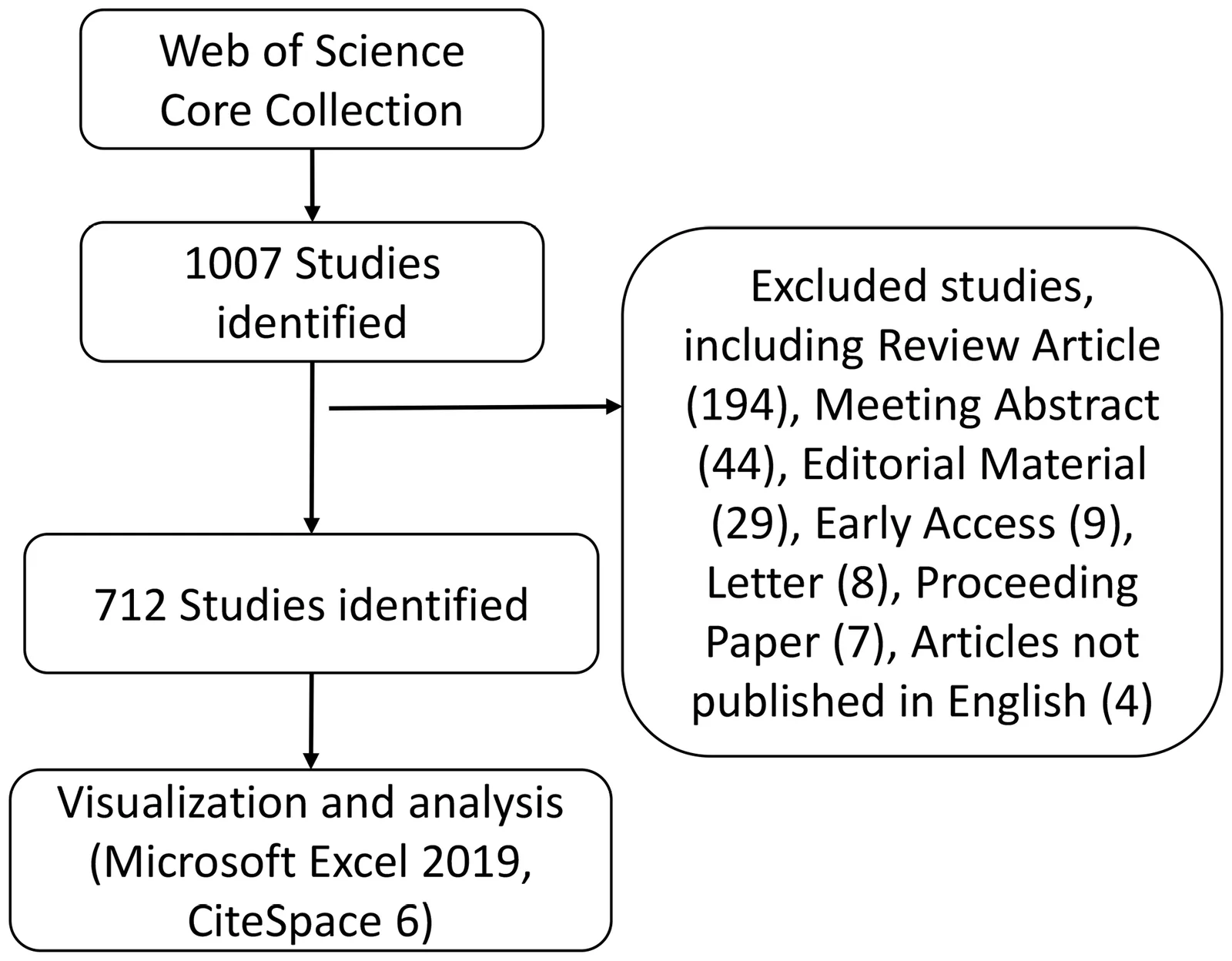
Flowchart of the literature selection.

### 2.2. Data analysis

This study employed Cite Space (version 6.3.R1) for bibliometric and visualization analyses. Developed by Professor Chaomei Chen at Drexel University, Cite Space is a Java-based application that utilizes data visualization techniques and scientometric methods to extract valuable information, including keyword co-occurrence, keyword clustering, journal co-citations, document co-citations, and author co-citations. The software provides timeline views that help to elucidate the current research landscape of a specific field and predict emerging research hotspots and trends.

Create a project titled WoS in Cite Space and configure the following parameters: Time slice set from January 2005 to October 2025, with 1 year for each slice. Select the top 10 as the criterion, Pruning: Pathfinder, cut the bidding network, and retain the remaining settings as default. Based on the research objectives, choose the node type fields of “country, institution, author, cited author, cited journal, cited reference, keyword.” From the submenu, select the keyword Burst Analysis.

Generally, each node on the map represents an author, keyword, or institution, with the size of the node indicating its frequency of occurrence or mention. The colored nodes correspond to specific years, while the colored circles extending outward from the nodes illustrate the time range from 2005 to 2025. In addition, the lines connecting nodes signify cooperative, coexistence, or co-entry relationships. Centrality is employed to assess the importance of nodes within the network; nodes exhibiting higher centrality indicate more frequent connections with other nodes, thereby suggesting their greater significance within the overall network. Betweenness centrality measures the degree to which a node resides on the shortest paths connecting other nodes. Nodes exhibiting high centrality (>0.1) are frequently regarded as “bridges” that enhance the flow of knowledge among research communities. Network density indicates the ratio of actual collaborations to all potential connections. This bibliometric analysis was conducted and reported in accordance with the Strengthening the Reporting of Observational Studies in Epidemiology (BIBLIO) statement.

## 3. Results

### 3.1. Publication trends

The initial search retrieved 1007 publications. After rigorous screening, 712 core publications were included in the bibliometric analysis. The annual distribution of publications serves as a key indicator of a field’s research activity and developmental stages. As shown in Figure 2, the annual publication output in the HCM-SCD field has demonstrated a significant upward trend from 2005 to 2025. The compound annual growth rate (CAGR) over the entire period was 10.8%, reflecting a consistent and ongoing increase in research activities. From 2005 to 2008, the annual number of publications remained relatively low, averaging 8.5 papers per year, which marked the initial stage of knowledge accumulation. Following 2010, a notable acceleration occurred, leading the field into a phase of rapid expansion. Importantly, after the release of the primary clinical guidelines, the published output exhibited a significant inflection point. In the five years preceding the issuance of the 2014 ESC guidelines (2009–2013), the average annual number of publications was 21.2; this figure rose to 39.4 per year in the subsequent 5 years (2015–2019), representing an increase of 85.85%. A further surge was noted surrounding the release of the 2020 AHA guidelines, with annual production peaking in 2021 at 66 articles. (Detailed data are shown in Supplementary Table S2, Supplemental Digital Content 2).

**Figure 2.**
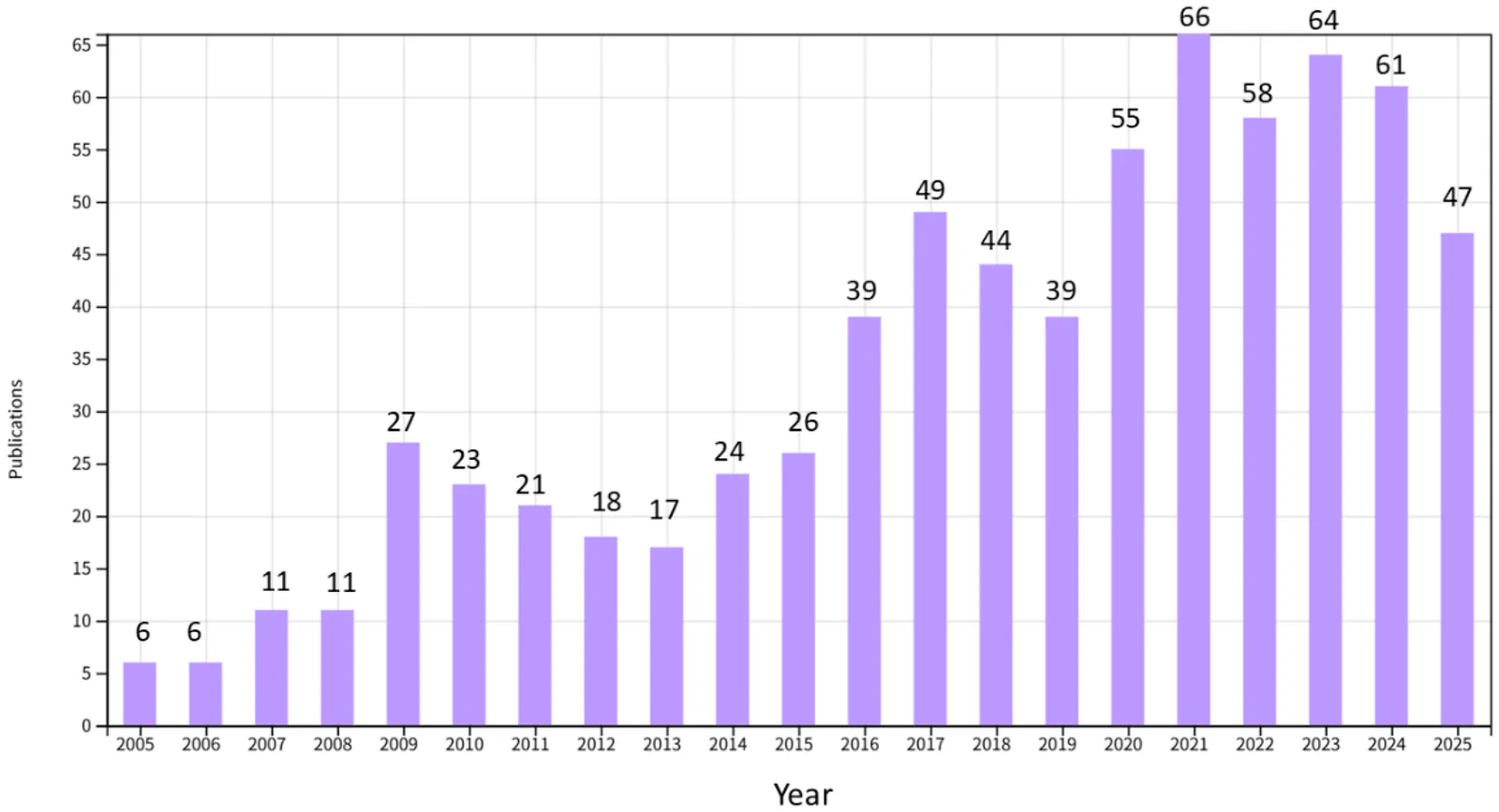
Publication trends in HCM-SCD research from 2005 to 2025. HCM-SCD = Hypertrophic cardiomyopathy-related sudden Cardiac death.

This trend closely aligns with the release and subsequent widespread discussion of the 2014 ESC and 2020 AHA/ACC guidelines, marking the entry of HCM-SCD research into a vigorous development period centered on evidence-based medicine and precise risk stratification. The slight decline in publications after 2021 may suggest a transition from rapid knowledge expansion to a phase of deepening, integration, and reflection within the field, or it could be related to the cutoff date for data retrieval (59.2 articles per year). This clear evolutionary trajectory not only reflects the growing academic attention to HCM-SCD but also implies the driving role of key guidelines, technological breakthroughs, and clinical needs behind it. Sustained growth in publication output establishes an active tone for the field, while the subsequent analysis of national and institutional collaboration networks will reveal the core forces and their geographical distribution driving this scientific growth.

### 3.2. Analysis of countries and institutions

At the national level (Table 1 and Figure 3), the United States (203 publications) held a dominant position in both publication count and betweenness centrality (0.40), highlighting its role not only as the most productive research entity but also as a vital hub within the global collaboration network. Although China ranked second in publication output (123 publications), its relatively low betweenness centrality (0.01) indicates the potential for expanding the scope and depth of its international collaborations, as its current research profile reflects a more independent system. Notably, European countries such as Italy and the United Kingdom exhibited higher betweenness centrality despite having lower publication volumes than the U.S. and China, underscoring their crucial role as “bridges” that connect different research communities and facilitate cross-regional knowledge exchange.

**Table 1:** Top 10 productive countries in HCM-SCD research.

No.	Country	Count	Centrality	Yr
1	United States of America	203	0.40	2005
2	Peoples R China	123	0.01	2007
3	Italy	90	0.17	2006
4	England	87	0.33	2006
5	Netherlands	53	0.05	2007
6	Germany	51	0.04	2005
7	Japan	44	0.01	2007
8	Canada	35	0.03	2011
9	Spain	33	0.03	2005
10	Australia	30	0.10	2006

**Figure 3.**
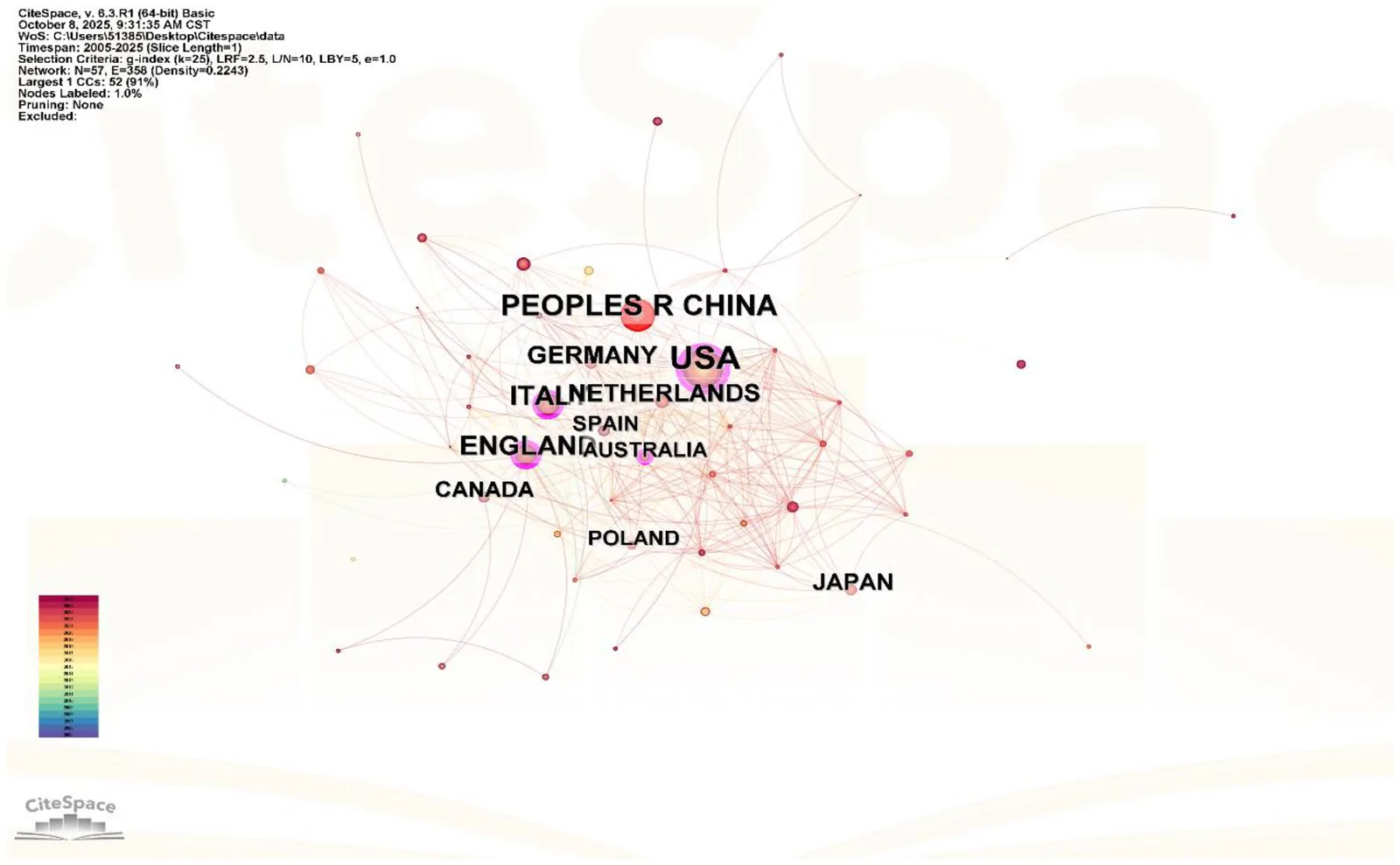
Collaborative network of countries/regions in HCM-SCD research. The nodes represent countries; the number of lines represents the intensity of cooperation between countries. Purple rings indicate high centrality. HCM-SCD = Hypertrophic cardiomyopathy-related sudden Cardiac death.

At the institutional level (Table 2 and Figure 4), high-yield institutions were primarily located in China, the United States, and Europe. A key finding was that publication volume did not directly correlate with influence. For example, institutions such as University College London (centrality 0.18) and Azienda Ospedaliero Universitaria Careggi (centrality 0.11), despite not having the highest publication counts, demonstrated substantial influence within the collaboration network owing to their high centrality. The institutional collaboration network density (0.022) is significantly lower than that of the national collaboration network (0.2243), indicating that cooperation occurs more frequently at the country level. Although transnational collaborations between institutions exist, they remain relatively sparse and present considerable potential for future growth.

**Table 2:** Top 10 productive institutions in HCM-SCD research.

No.	Institution	Count	Centrality	Yr
1	Chinese academy of medical sciences	42	0.01	2013
2	Fu Wai hospital Chinese academy of medical sciences	42	0.01	2013
3	University of London	39	0.03	2006
4	Peking union medical college	39	0.00	2014
5	Harvard university	34	0.08	2008
6	Azienda Ospedaliero Universitaria Careggi	34	0.11	2009
7	Harvard university medical affiliates	33	0.07	2008
8	University college London	33	0.18	2006
9	University of florence	33	0.05	2016
10	Mayo clinic	29	0.07	2005

**Figure 4.**
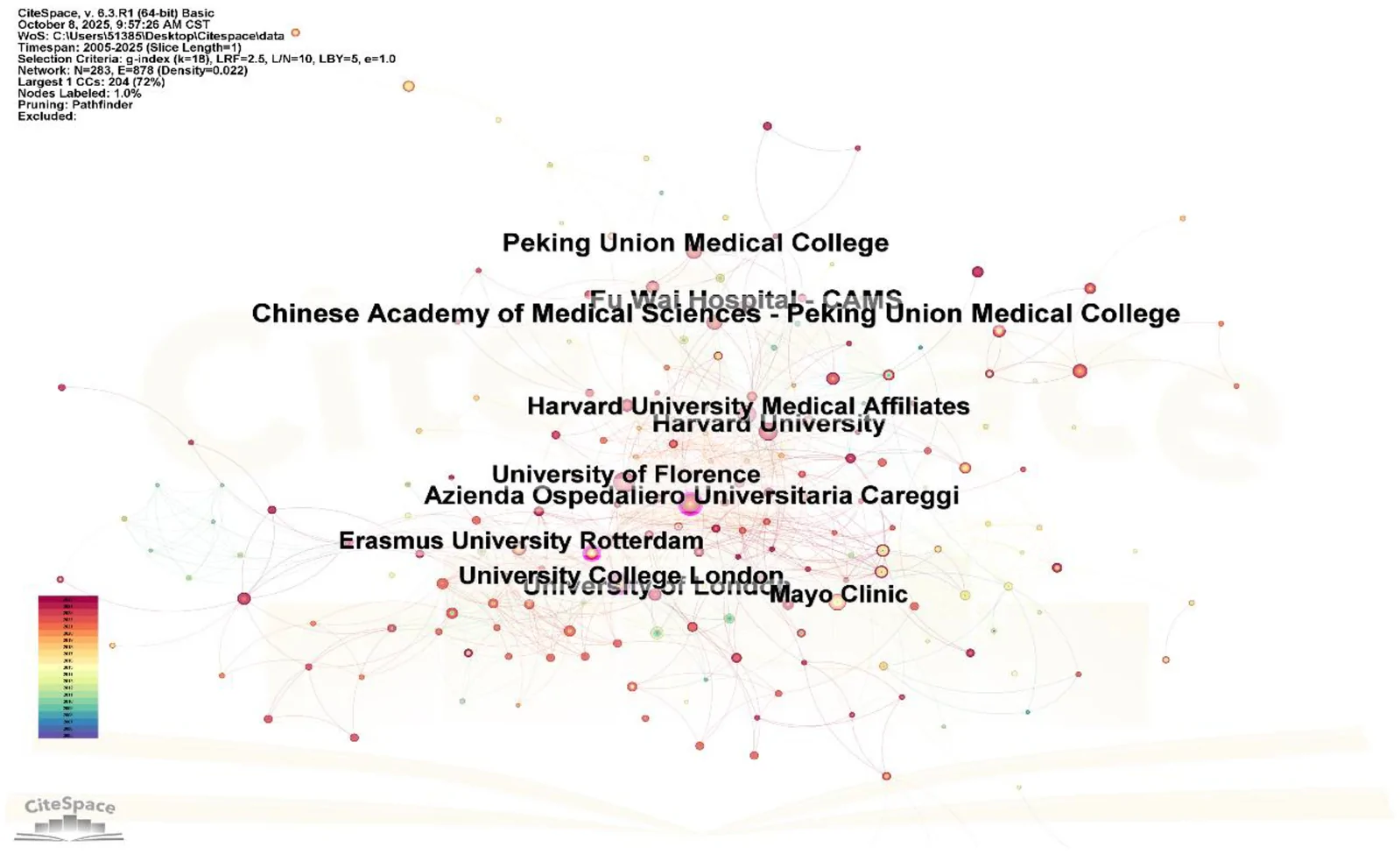
Collaborative Network of Institutions for HCM-SCD Research. The nodes represent institutes; the number of lines represents the intensity of cooperation between institutions. Purple rings indicate high centrality. HCM-SCD = Hypertrophic cardiomyopathy-related sudden Cardiac death.

### 3.3. Analysis of co-cited journals

Journal co-citation analysis revealed the knowledge sources and disciplinary foundations of the HCM-SCD research field. Figure 5 illustrates the distribution of the most frequently cited journals in this domain, identifying Circulation (cited 678 times), Journal of the ACC (cited 662 times), and European Heart Journal (cited 573 times) as the 3 most frequently cited journals, forming the core knowledge base for HCM-SCD research. This finding indicates that the field relies heavily on the high-impact evidence published in top-tier cardiovascular medicine journals, thereby establishing a robust and authoritative knowledge foundation (Table 3).

**Table 3:** Top 10 productive cited journal in HCM-SCD research.

No.	Count	Centrality	Yr	Cite Journal
1	678	0.00	2005	Circulation
2	662	0.01	2005	Journal of the American college of cardiology
3	573	0.03	2005	European heart journal
4	456	0.02	2005	American journal of cardiology
5	431	0.01	2005	Heart
6	405	0.00	2005	New England journal of medicine
7	270	0.02	2005	Journal of the American Medical Association
8	254	0.01	2005	The Lancet
9	216	0.10	2006	International journal of cardiology
10	202	0.04	2009	Circulation-heart failure

**Figure 5.**
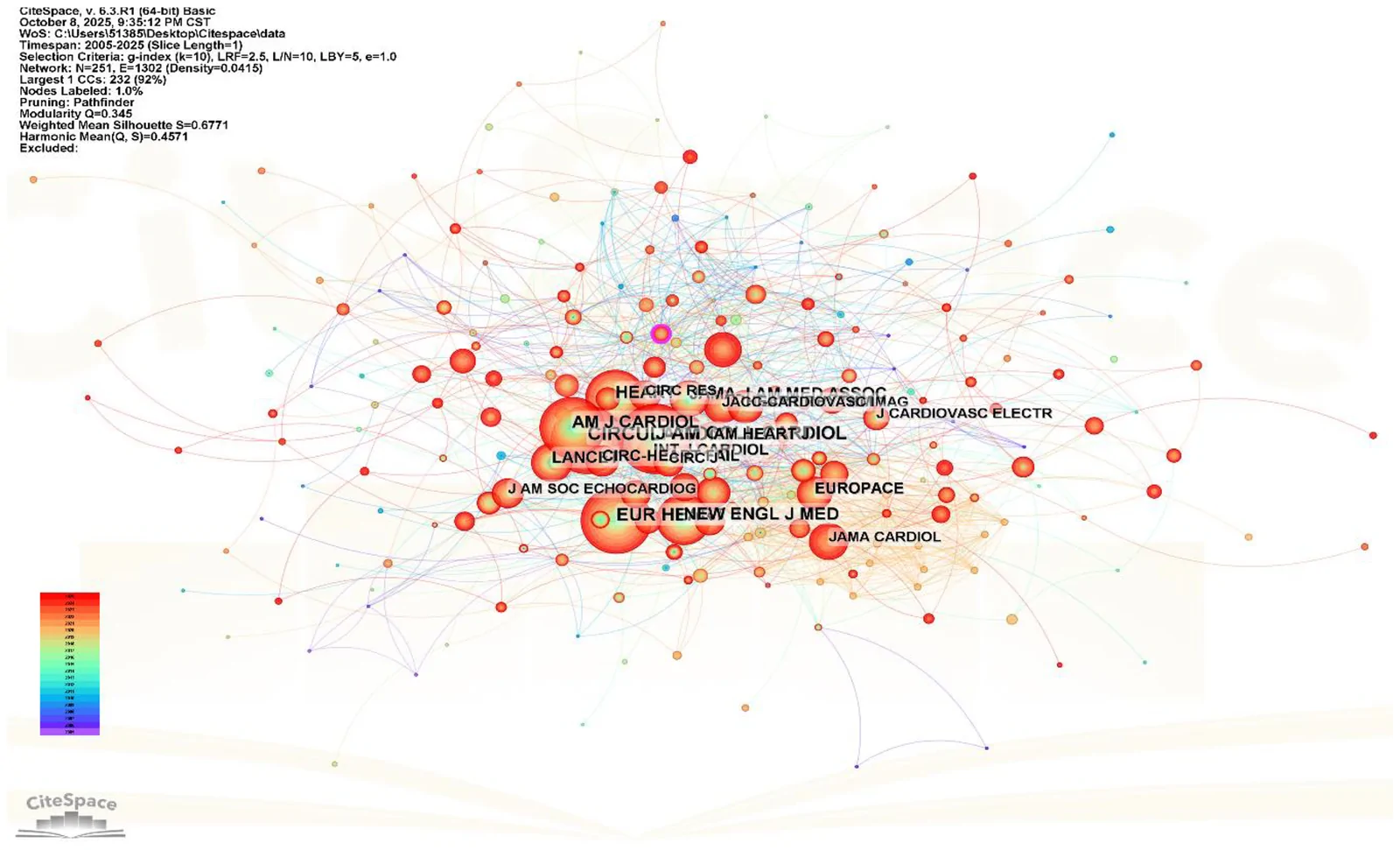
Co-citation network of journals in HCM-SCD research. The nodes in the map represent journals, and lines between the nodes represent co-journal relationships. Purple rings indicate high centrality. HCM-SCD = Hypertrophic cardiomyopathy-related sudden Cardiac death.

### 3.4. Analysis of authors and co-cited authors

A total of 4837 authors contributed to the publications on HCM-SCD (see Table 4). The most prolific author was Olivotto, I from the United States, with 23 articles, followed by Maron, MS (21 articles), Maron, BJ (15 articles), and Ommen, SR (15 articles). Notably, these authors exhibited relatively high betweenness centrality values (>0.01), indicating their substantial and increasing influence in SCD research. In the visualization (Fig. 6), each circle represents an author, and the circle size corresponds to the number of publications. Thicker lines between circles indicate stronger collaborative relationships, whereas different colors represent distinct collaborative groups. Authors who were frequently cited together in subsequent publications were identified as co-cited authors (Fig. 7). Among the 673 co-cited authors, seven were cited more than 150 times. This cohort of productive and highly cited authors fundamentally shaped the foundational knowledge of the field.

**Table 4:** Top 9 authors and co-cited authors in HCM-SCD research.

No.	Count	Centrality	Yr	Author	No.	Count	Centrality	Yr	Co‐cited author
1	23	0.15	2016	Olivotto, I	1	576	0.07	2005	Maron, BJ
2	21	0.03	2015	Maron, MS	2	413	0.15	2006	Elliott, PM
3	15	0.03	2008	Maron, BJ	3	264	0.00	2012	Gersh, BJ
4	15	0.04	2010	Ommen, SR	4	244	0.26	2008	Maron, MS
5	14	0.03	2007	Ackerman, MJ	5	224	0.04	2014	Omahony, C
6	14	0.05	2013	Semsarian, C	6	183	0.00	2007	Olivotto, I
7	13	0.05	2015	Link, MS	7	179	0.24	2007	Ommen, SR
8	10	0.07	2011	Michels, M	8	148	0.34	2005	Spirito, P
9	9	0.02	2009	Elliott, PM	9	119	0.08	2005	Ho, CY

**Figure 6.**
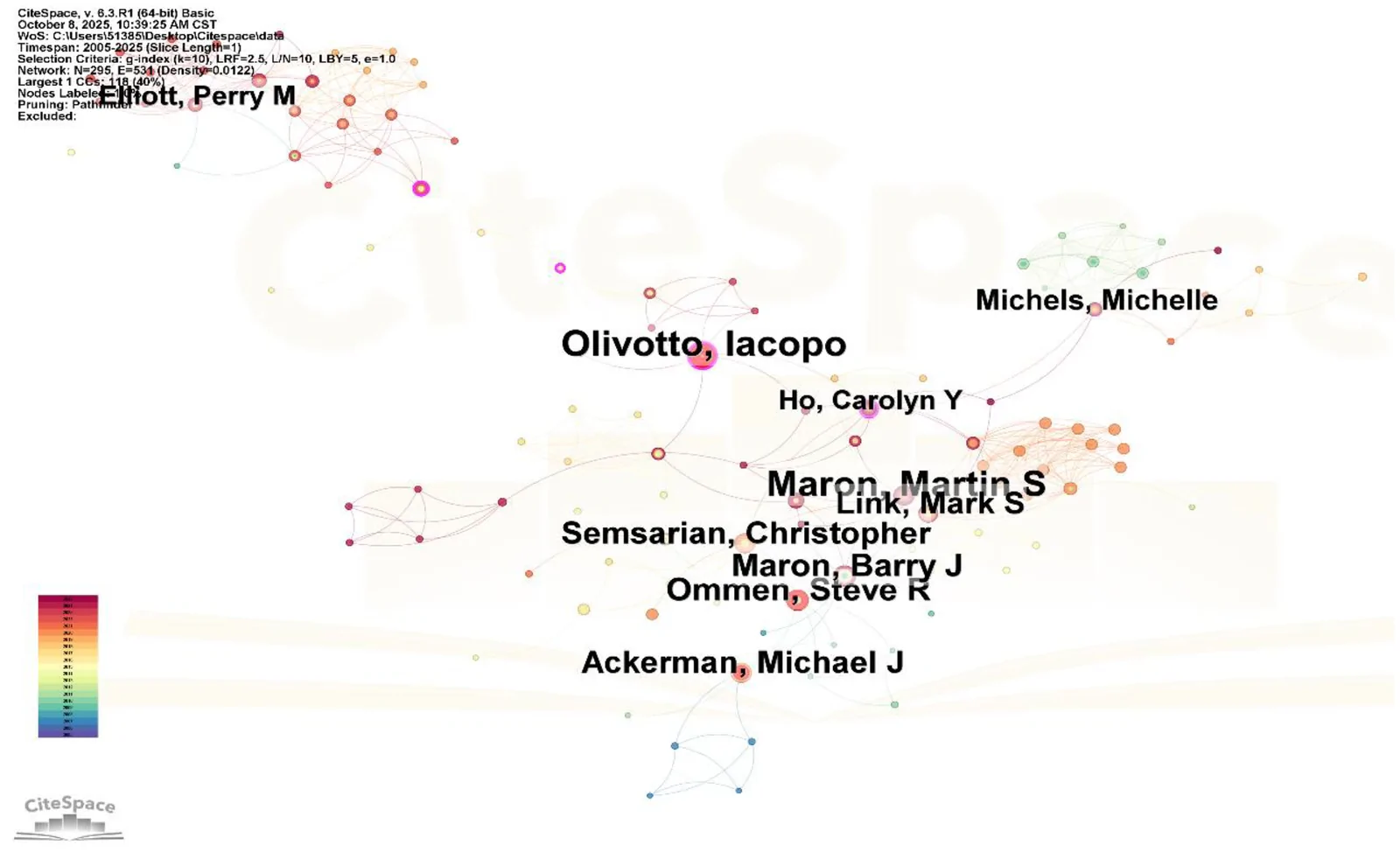
Collaboration network of authors in HCM-SCD research. The nodes in the map represent authors, and lines between the nodes represent co-citation relationships. Purple rings indicate high centrality. HCM-SCD = Hypertrophic cardiomyopathy-related sudden Cardiac death.

**Figure 7.**
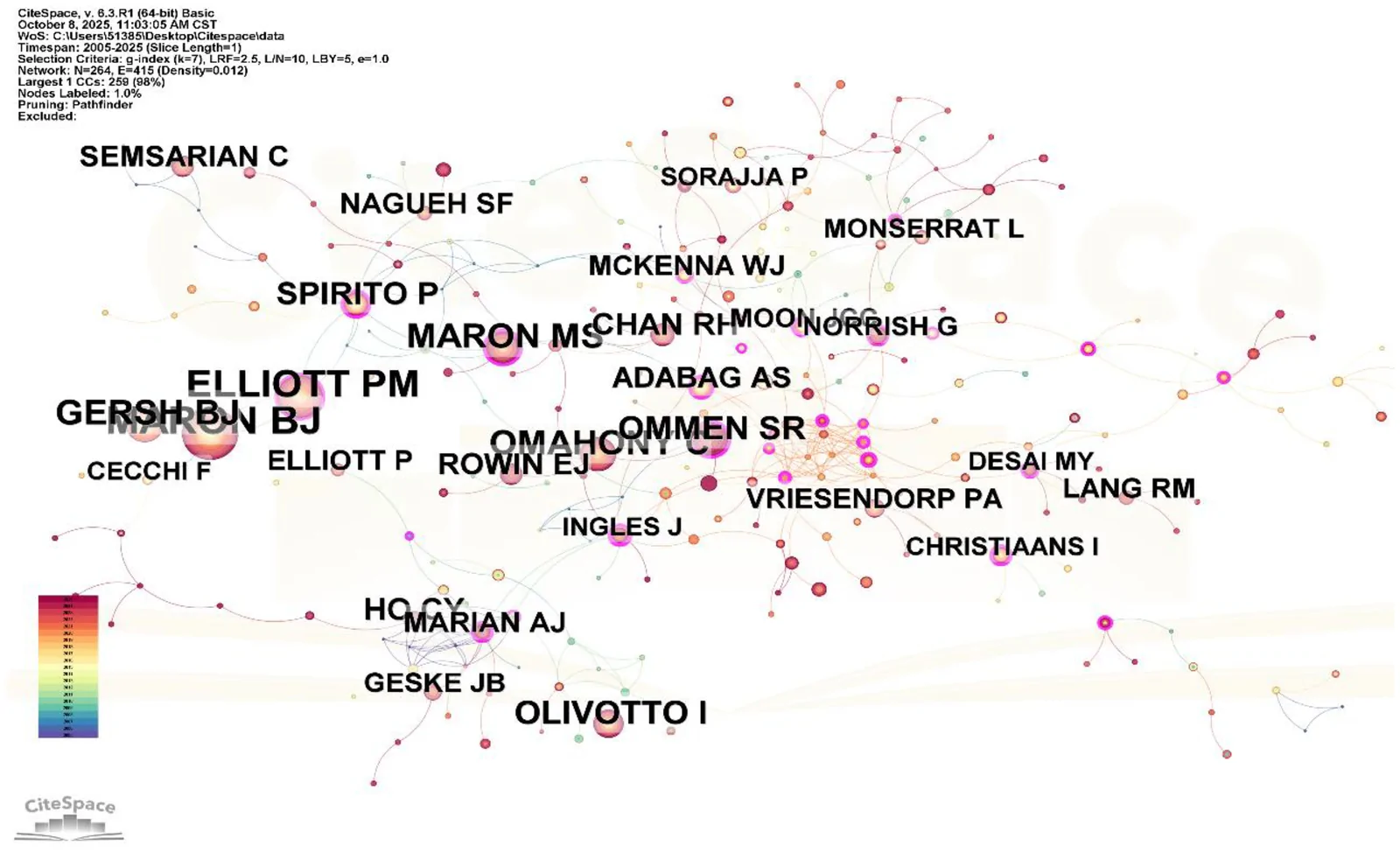
Co-citation network of authors in HCM-SCD research. The nodes in the map represent coauthors, and lines between the nodes represent co-citation relationships. Purple rings indicate high centrality. HCM-SCD = Hypertrophic cardiomyopathy-related sudden Cardiac death.

### 3.5. Analysis of co-cited references

Co-cited references indicate that these publications share intellectual value and influence within a specific research domain, serving as a key metric for assessing the academic impact of the literature. We identified the top 10 most frequently cited references in HCM-SCD research (Table 5). Notably, five of these ten references are international authoritative guidelines (ESC, AHA/ACC), strongly demonstrating that this field is highly standardized and guideline-driven. Clinical practice guidelines (e.g., the 2014 ESC and 2020 AHA/ACC guidelines) not only inform clinical decision-making but also provide a theoretical framework for researchers designing trials and selecting endpoints. Concurrently, risk prediction models (e.g., the 2014 HCM risk-SCD model) and prognostic validation studies of key technologies (e.g., the prognostic value of Late Gadolinium Enhancement [LGE]-Cardiac Magnetic Resonance ) collectively form the evidential foundation supporting these guideline recommendations.

**Table 5:** Top 10 co-cited references in HCM-SCD research.

No.	Reference	Count	Centrality	Yr
1	2014 ESC Guidelines on diagnosis and management of hypertrophic cardiomyopathy: the task force for the diagnosis and management of hypertrophic cardiomyopathy of the European society of cardiology (ESC)	115	0.33	2014
2	A novel clinical risk prediction model for sudden cardiac death in hypertrophic cardiomyopathy (HCM risk-SCD)	63	0.51	2014
3	Enhanced American college of cardiology/American heart association strategy for prevention of sudden cardiac death in high-risk patients With hypertrophic cardiomyopathy	48	0.10	2019
4	2020 AHA/ACC guideline for the diagnosis and treatment of Patients with hypertrophic cardiomyopathy: executive summary: A report of the American college of cardiology/American Heart association joint committee on clinical practice guidelines	43	0.00	2020
5	Prognostic value of quantitative contrast-enhanced cardiovascular magnetic resonance for the evaluation of sudden death risk in patients with hypertrophic cardiomyopathy	39	0.16	2014
6	2023 ESC guidelines for the management of cardiomyopathies	38	0.03	2023
7	Implantable cardioverter-defibrillators and prevention of sudden cardiac death in hypertrophic cardiomyopathy	36	0.19	2007
8	2020 AHA/ACC Guideline for the diagnosis and treatment of patients with hypertrophic cardiomyopathy: A report of the American college of cardiology/American heart association joint committee on clinical practice guidelines	36	0.05	2020
9	International external validation study of the 2014 European society of cardiology guidelines on sudden cardiac death prevention in hypertrophic cardiomyopathy (EVIDENCE-HCM)	36	0.27	2018
10	Genotype and lifetime burden of disease in hypertrophic cardiomyopathy: insights from the Sarcomeric human cardiomyopathy Registry (SHaRe)	35	0.12	2018

### 3.6. Keyword and keyword burst analysis

Keyword analysis offers a macroscopic perspective of the structure and dynamics of research topics. High-frequency keywords (Table 6), such as “HCM,” SCD, “risk stratification,” and “guidelines,” corroborate the findings from the co-citation analysis, reaffirming the core themes of the field. Using Cite Space, keyword clustering analysis was conducted to produce the visual map shown in Figure 8. This map groups high-frequency keywords into 4 major thematic clusters: the red cluster centers on “HCM,” the yellow on “SCD,” the green on “late gadolinium enhancement,” and the blue on “risk stratification.” Keyword burst analysis (Fig. 9) serves as a “radar” for the field, detecting emerging or declining themes. The progression of burst keywords from early terms like “cytochrome c oxidase, mechanistic exploration, to recent terms such as “machine learning” and “mavacamten” vividly illustrates the field’s historical shift in research paradigms, moving from basic biology to data science and targeted therapy. S mole T et al (2021) have shown that machine learning, specifically utilizing gradient boosting trees, outperforms conventional risk stratification tools in forecasting the combined occurrence of ventricular arrhythmias, such as SCD.^[10]^ This advancement gained traction subsequent to the publication of the 2020 ACC guidelines, which significantly broadened the theoretical basis for risk evaluation. The guidelines moved away from a simplistic tally of risk factors towards a holistic, multifaceted, and personalized strategy. This evolution has cultivated a setting that is favorable for the integration of machine learning techniques into clinical practice.

**Table 6:** Top 10 keywords by frequency in HCM-SCD research.

No.	Keywords	Count	Centrality	Yr
1	Hypertrophic cardiomyopathy	626	0.10	2005
2	Sudden cardiac death	347	0.24	2005
3	Diagnosis	159	0.06	2013
4	Task force	156	0.04	2010
5	Sudden death	120	0.11	2005
6	Risk	108	0.02	2008
7	European society	103	0.08	2009
8	Management	100	0.05	2010
9	Risk stratification	91	0.06	2010
10	Guidelines	89	0.02	2013

**Figure 8.**
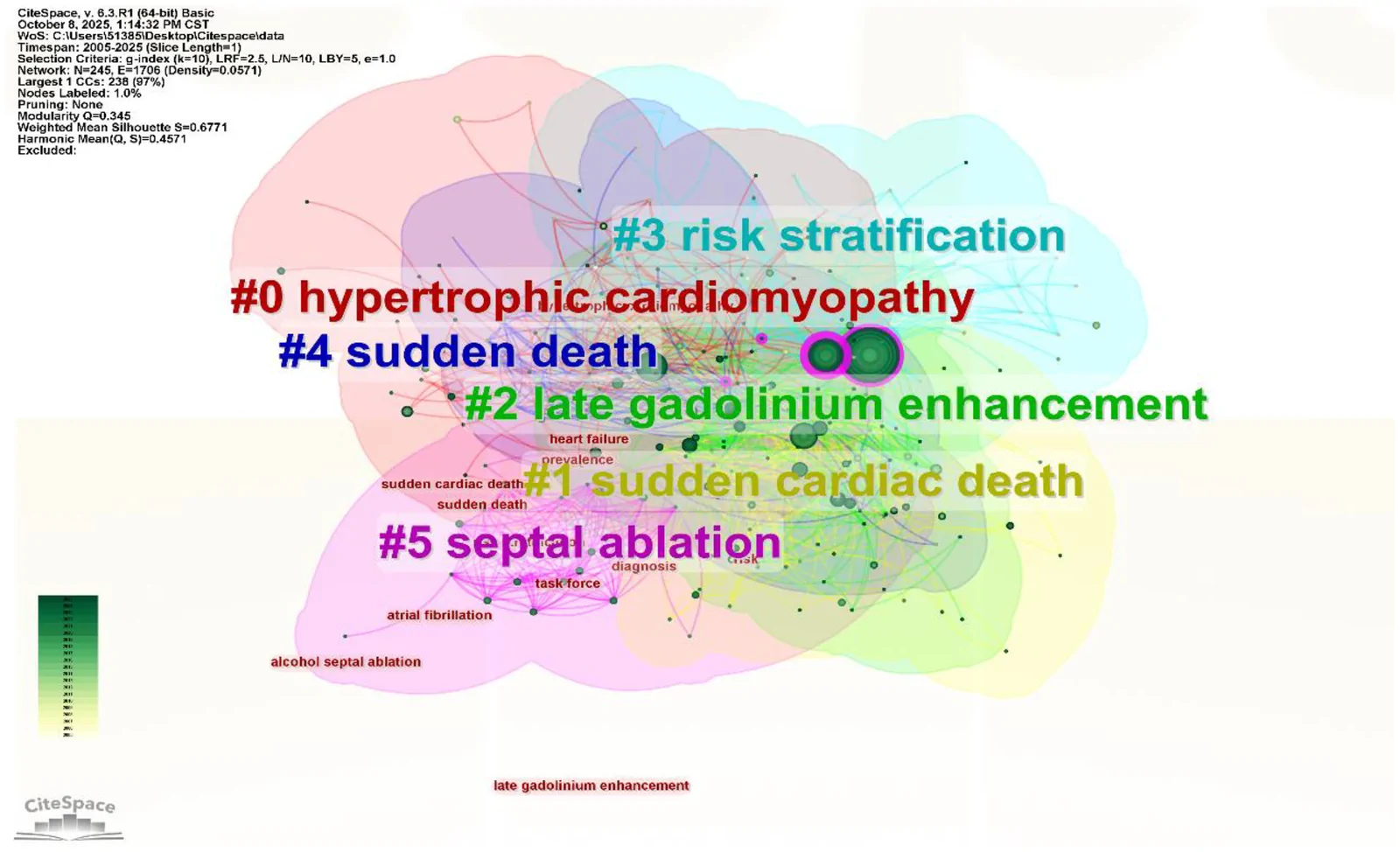
Keyword co-occurrence clusters in HCM-SCD research. HCM-SCD = Hypertrophic cardiomyopathy-related sudden Cardiac death.

**Figure 9.**
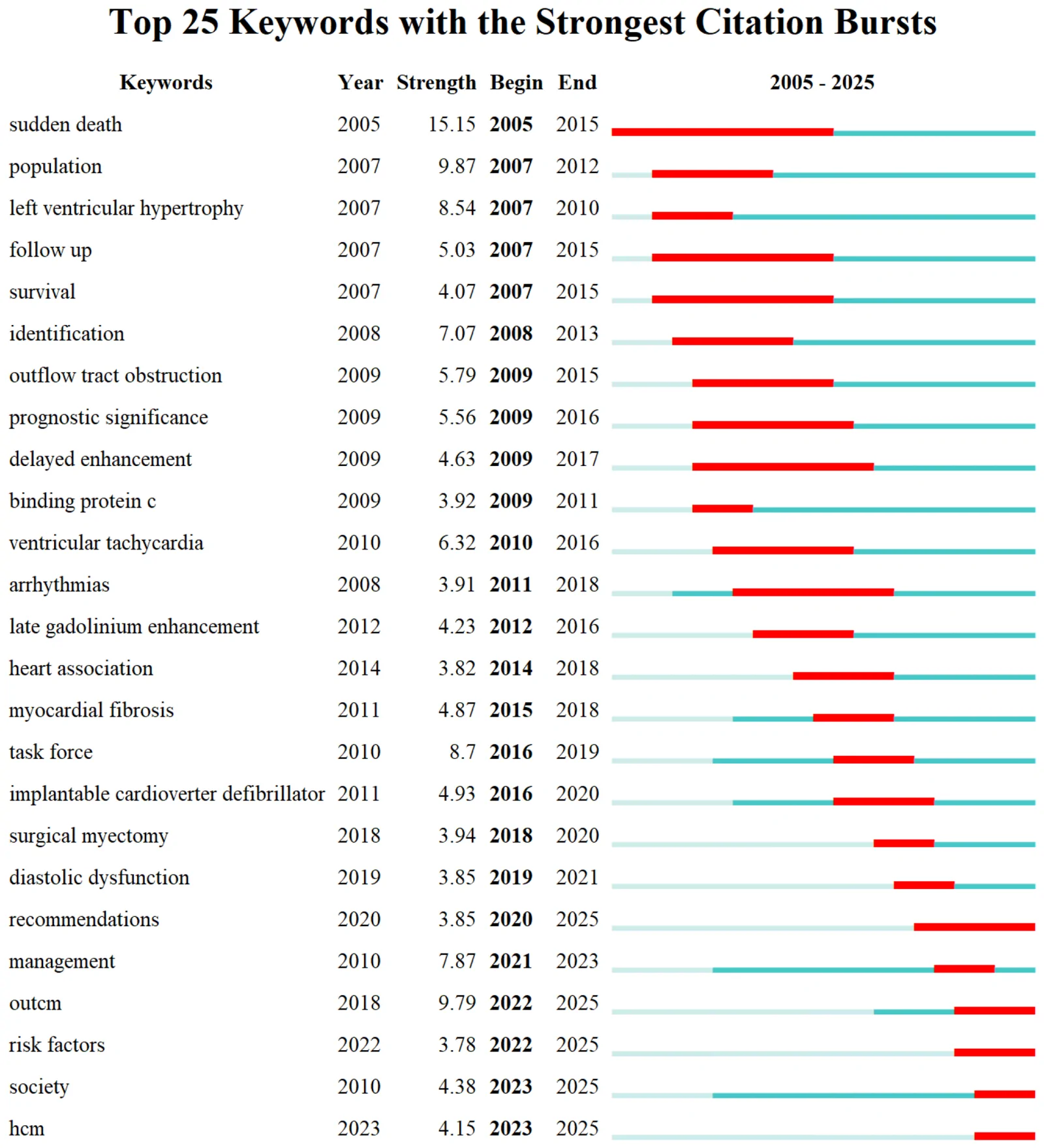
The top 25 keywords with the strongest citation bursts in HCM-SCD research. The red horizontal stripes represent the years with the most frequent keywords. The green horizontal stripes represent the years with the most infrequent keywords. HCM-SCD = Hypertrophic cardiomyopathy-related sudden Cardiac death.

### 3.7. Timeline analysis

The timeline visualization in this study (Fig. 10) clearly illustrates 4 distinct evolutionary phases in HCM-SCD research paradigms. During the initial phase (2005–2010), research focused on disease definition, diagnosis, and epidemiology. This period was characterized by keywords such as “SCD,” “HCM,” “diagnosis,” “prevalence,” “magnetic resonance,” “sudden death,” and “ventricular fibrillation.” The period from 2011 to 2016 marked the core phase of risk stratification, during which implantable cardioverter-defibrillators became the central intervention strategy. The integration of genetics and LGE as novel biomarkers, alongside traditional risk factors, has signaled the advent of multimodal risk assessment. Key terms included “risk stratification,” “implantable cardioverter defibrillator,” “LGE,” “alcohol septal ablation,” and “task force.” From 2017 to 2020, the application of artificial intelligence and machine learning marked a shift toward nonlinear, high-dimensional, data-driven risk prediction models. The emphasis on shared decision-making reflects a growing commitment to patient-centered, individualized care. Representative keywords included “prediction model,” “surgical myectomy vs septal ablation,” “exercise stress testing,” and “subclinical atrial fibrillation.” The most recent phase (2021–2025) has witnessed the emergence of cardiac myosin inhibitors, such as mavacamten, marking a paradigm shift from symptomatic management (relieving obstruction) to targeting the underlying disease mechanisms by modulating hyper contractility. Key terms from this period include “mavacamten,” “machine learning/AI,” “global longitudinal strain (GLS),” “cardiac myosin inhibitor,” “primary prevention,” “pediatric cardiomyopathy/childhood,” and “energetics/apoptosis.”

**Figure 10.**
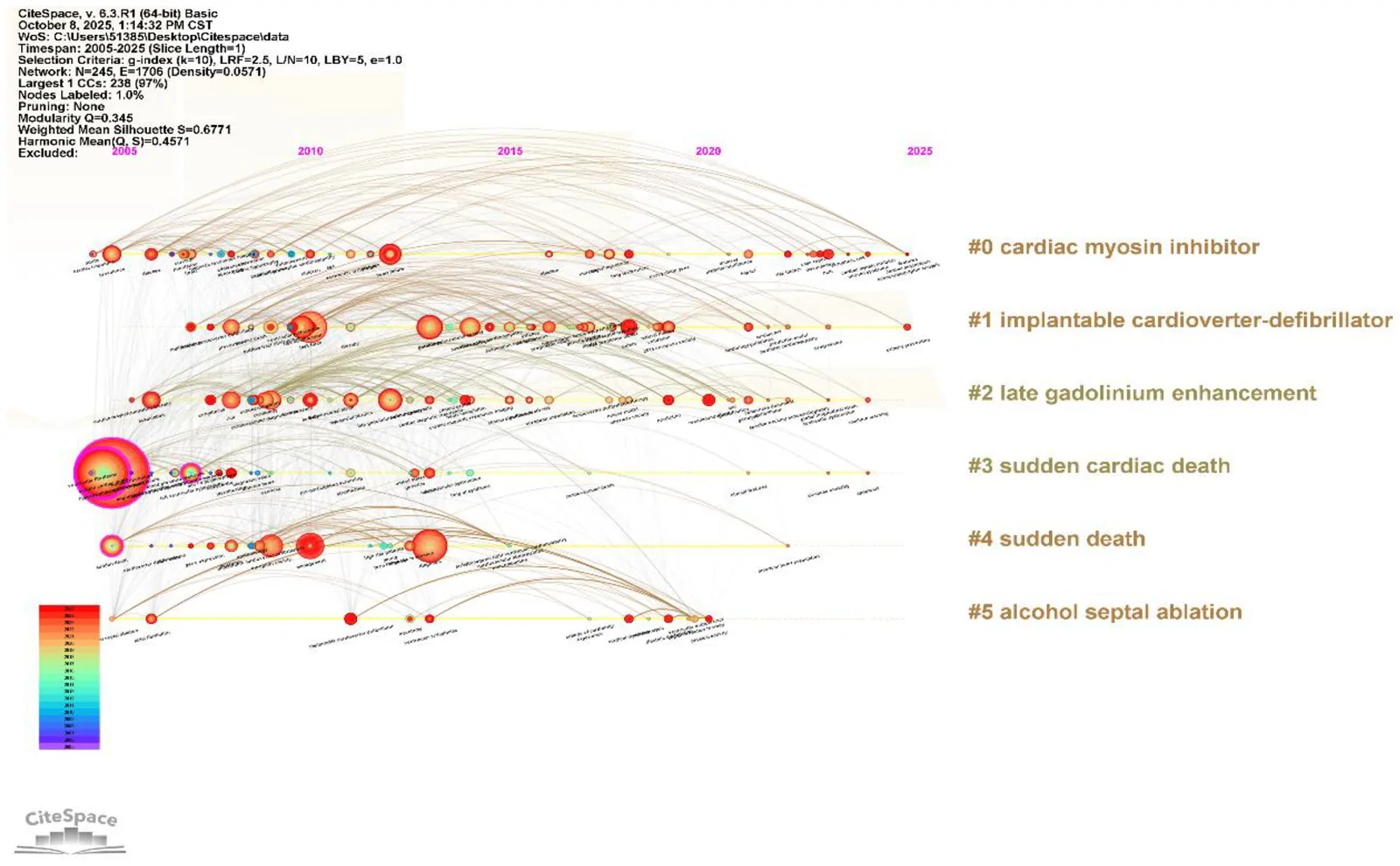
Timeline view of theme evolution in HCM-SCD research. HCM-SCD = Hypertrophic cardiomyopathy-related sudden cardiac death.

## 4. Discussion

This study presents the first comprehensive bibliometric analysis of research literature in the field of HCM-SCD over the past 21 years (2005–2025). It uncovers the cooperation network, knowledge base, hotspot evolution, and development trends in this area, offering clinicians and researchers a macro perspective and practical insights.

### 4.1. Global collaboration networks and distribution of research forces

The study identified a significant growth trend in the HCM-SCD research field, with a compound annual growth rate of 10.8% from 2005 to 2025. Notably, this growth accelerated following the release of the 2014 ESC guidelines and the 2020 AHA/ACC guidelines, underscoring the “guide-driven” development characteristic of this field. In a national-level analysis, the United States stands out with 203 publications and a betweenness centrality of 0.40, underscoring its position as both the most prolific research entity and the central hub of the global cooperation network. While European countries like Italy (centrality 0.17) and the United Kingdom (centrality 0.33) have fewer publications than the United States and China, their significant centrality underscores their pivotal role in linking diverse research groups and fostering cross-regional knowledge exchange. This indicates that extensive international collaboration is vital for enhancing research impact and facilitating knowledge dissemination. China ranks second in the number of publications (123) but has a low betweenness centrality of 0.01, suggesting ample opportunity to broaden its international collaboration. This aligns with China’s current stage of being “big but not strong” in scientific research. Moving forward, Chinese scholars should engage more actively in international multi-center research and academic exchanges, transitioning from “independent output” to “deep integration” to boost their global influence and academic discourse. At the institutional level, high-output institutions are primarily located in China, the United States, and Europe.

### 4.2. Knowledge base and research hotspot evolution

The co-citation analysis results indicate that the knowledge base in the field of HCM-SCD is heavily concentrated on international authoritative clinical guidelines and risk prediction models. Among the top ten co-cited articles, five are clinical guidelines issued by ESC or AHA/ACC, while four are risk prediction models or validation studies based on these guidelines. This strongly supports the “guide-driven” nature of this field. Notably, the 2014 ESC guidelines introduced cardiac magnetic resonance LGE as an independent risk marker and the HCM Risk-SCD model, marking a shift from traditional “risk factor count” to “quantitative risk assessment.”^[3,11,12]^

Keyword analysis reveals the dynamic evolution of research focus within this field. High-frequency keyword clustering has formed 4 major thematic clusters: disease entity (HCM), clinical outcome SCD, assessment tool LGE, and prevention strategy (risk stratification).^[13,14]^ This structure demonstrates the field’s comprehensive knowledge chain, progressing from “disease identification” to “risk quantification” and “intervention strategy.” The timeline map (Fig. 10) further details this evolution. In the early stage (2005–2010), research concentrated on disease definition, diagnosis, and epidemiological characteristics. Between 2011 and 2016, risk stratification became the central theme, with ICD implantation as the primary intervention method, and LGE and genetics were incorporated into the multimodal risk assessment framework as new biomarkers.^[15–17]^ From 2017 to 2020, the integration of artificial intelligence and machine learning signifies a transformation towards nonlinear, high-dimensional, data-driven models. Notably, “risk factor” emerged as a significant keyword during 2014 to 2020.

### 4.3. Emerging directions and future prospects

As the knowledge system in the field of HCM-SCD matures, research is advancing in 3 interconnected directions. First, risk stratification is becoming more intelligent. The advent of machine learning and artificial intelligence, with “machine learning” emerging as a key term in 2020, is transforming risk prediction paradigms. Smole et al^[10]^ demonstrated that a machine learning model based on the lifting tree algorithm significantly outperformed traditional risk stratification tools in predicting the composite endpoint of ventricular arrhythmia. This shift indicates a move from the “simple superposition of several risk factors” to the “complex integration of multi-dimensional data.” In the future, dynamic risk prediction models that integrate clinical, imaging, genetic, and biomarker data are expected to enable truly individualized SCD prevention. Secondly, there is a focus on the etiology of treatment strategies. Finally, clinical management is becoming more individualized. The recent emphasis on “shared decision-making” reflects a shift in clinical practice from “one-size-fits-all” guideline recommendations to “patient-centered” individualized regimens^.[18]^ This approach requires a careful balance of the benefits and risks of ICD implantation, taking into account patients’ quality of life and value orientations. Additionally, the treatment of left ventricular outflow tract obstruction whether through medication, intervention, or surgery necessitates precise selection based on the patient’s anatomical structure and pathophysiological characteristics.^[19–22]^

### 4.4. Advantages and limitations of the research

This paper has several advantages: it is the first systematic bibliometric analysis on HCM-SCD and fills a significant gap. The study spans 21 years, from 2005 to 2025, so it can be studied in detail. CiteSpace multidimensional analysis yields systematic and more detailed results. Moreover, its inclusion in the early access literature makes the work relevant and timely. This study has some limitations. First, the data sources are limited to the WoS Core Collection, which is a preferred source for bibliometric analysis but may overlook non-English literature and studies only available in Scopus and PubMed. Second, although early access 2025 was considered, it may not fully represent the annual publication volume and may affect trend analysis for the last year. Third, bibliometric methods focus on quantitative analysis, revealing knowledge structures and trends but not entirely capturing the complexity of scientific discourse and qualitative research aspects. Fourth, non-research literature such as guidelines and editorials were excluded, which could underestimate their influence, although indirectly reflected in co-citation analysis.

## 5. Conclusion

These findings indicate that the field has matured into a well-established research domain, characterized by a core foundation of guideline consensus, a framework for multimodal risk assessment, and a basis for imaging and genetics. Future research is expected to increasingly focus on the intellectualization of risk prediction, etiological targeting of treatment strategies, and personalization of management plans.

## Author contributions

**Conceptualization:** Yu Li, Fen Ai.

**Data curation:** Yu Li, Zhen Chen, Jinlei Li.

**Methodology:** Yu Li, Zhen Chen, Fen Ai.

**Writing – review & editing:** Fen Ai.




