# Supplementary material for: Research trends and hotspot analysis of sudden cardiac death in hypertrophic cardiomyopathy: A bibliometric analysis
Source: Medicine (Baltimore). 2026 Oct 23;105(43):e51272. doi: 10.1097/MD.0000000000051272 (PMC13706386; doi:10.1097/MD.0000000000051272)
Supplement: Supplementary file 1 [file medi-105-e51272-s001.docx]

Table S1: Supplementary.

TI = ("Cardiomyopathy, Hypertrophic" OR "Cardiomyopathies, Hypertrophic" OR "Hypertrophic Cardiomyopathies" OR "Hypertrophic Cardiomyopathy" OR "Cardiomyopathy, Hypertrophic Obstructive" OR "Cardiomyopathies, Hypertrophic Obstructive" OR "Hypertrophic Obstructive Cardiomyopathies" OR "Hypertrophic Obstructive Cardiomyopathy" OR "Obstructive Cardiomyopathies, Hypertrophic" OR "ObstructiveCardiomyopathy, Hypertrophic") AND AK = ("Cardiomyopathy, Hypertrophic" OR "Cardiomyopathies, Hypertrophic" OR "Hypertrophic Cardiomyopathies" OR "Hypertrophic Cardiomyopathy" OR "Cardiomyopathy, Hypertrophic Obstructive" OR "Cardiomyopathies, Hypertrophic Obstructive" OR "Hypertrophic Obstructive Cardiomyopathies" OR "Hypertrophic Obstructive Cardiomyopathy" OR "Obstructive Cardiomyopathies, Hypertrophic" OR "ObstructiveCardiomyopathy, Hypertrophic") AND TS=("Sudden Cardiac Death" OR "SCD" OR "cardiac arrest" OR "ventricular arrhythmia" OR "cardiogenic sudden death" OR "sudden cardial death" OR "sudden cardia death" OR "suddenly death of heart source"OR "sudden cardiac ").
