# Supplementary material for: Research trends and hotspot analysis of sudden cardiac death in hypertrophic cardiomyopathy: A bibliometric analysis
Source: Medicine (Baltimore). 2026 Oct 23;105(43):e51272. doi: 10.1097/MD.0000000000051272 (PMC13706386; doi:10.1097/MD.0000000000051272)
Supplement: Supplementary file 2 [file medi-105-e51272-s002.docx]

Supplementary Table S2. Annual publication output of HCM-SCD research

| Year | Number of Publications |
| --- | --- |
| 2005 | 6 |
| 2006 | 6 |
| 2007 | 11 |
| 2008 | 11 |
| 2009 | 27 |
| 2010 | 23 |
| 2011 | 21 |
| 2012 | 18 |
| 2013 | 17 |
| 2014 | 24 |
| 2015 | 26 |
| 2016 | 39 |
| 2017 | 49 |
| 2018 | 44 |
| 2019 | 39 |
| 2020 | 55 |
| 2021 | 66 |
| 2022 | 58 |
| 2023 | 64 |
| 2024 | 61 |
| 2025 | 47 |
